# The Initial Vascular Access Type Contributes to Inflammation in Incident Hemodialysis Patients

**DOI:** 10.1155/2012/917465

**Published:** 2011-10-25

**Authors:** Mala Sachdeva, Adriana Hung, Oleksandr Kovalchuk, Markus Bitzer, Michele H. Mokrzycki

**Affiliations:** ^1^Division of Nephrology, Department of Medicine, Montefiore Medical Center, Albert Einstein College of Medicine, 3332 Rochambeau Avenue, Centennial Building Room 423, Bronx, NY 10467, USA; ^2^Division of Nephrology, Department of Medicine, Vanderbilt University, Nashville, TN 37232, USA

## Abstract

*Background*. The contribution of the hemodialysis (HD) vascular access type to inflammation is unclear. *Methods*. We conducted a prospective observational study in an incident HD population. C-reactive protein (CRP), interleukin-6 (IL-6), and interferon-*γ*-induced protein (IP-10) were measured before and at 6-time points after access placement for 1 year. *Results*. Sixty-four incident HD patients were included (tunneled catheter (TC), *n* = 40, arteriovenous fistula (AVF), *n* = 14, and arteriovenous graft (AVG), *n* = 10). A mixed effects model was performed to adjust for age, sex, race, coronary artery disease, diabetes mellitus, infections, access thrombosis, initiation of HD, and days after access surgery. In comparison to AVFs, the presence of a TC was associated with significantly higher levels of CRP (*P* = 0.03), IL-6 (*P* = 0.07), and IP-10 (*P* = 0.03). The presence of an AVG was associated with increases in CRP (*P* = 0.01) and IP-10 (*P* = 0.07). *Conclusions*. Patients who initiate HD with a TC or an AVG have a heightened state of inflammation, which may contribute to the excess 90-day mortality after HD initiation.

## 1. Introduction

The most recent 2009 USRDS report observed high first- and second-month death rates after HD initiation [1]. The type of HD vascular access is significantly associated with risk of death. The CHOICE study reported 50% higher mortality rates in patients initiating HD with a TC as compared to patients with an AVF. There was a trend toward higher mortality rates (21% increased) in incident HD patients with an AVG compared to those with an AVF; however this did not achieve statistical significance [2]. A recently published Canadian study in 40,526 incident dialysis patients reported an 80% higher 1-year mortality for HD patients with a TC compared to PD patients and HD patients with an AVF or AVG [3]. Potential reasons for these findings include the higher rate of infection and sepsis associated with TCs, relative to AVFs; however the contribution of the vascular access type, independent of infection, may also be a factor. 

The prevalence of chronic inflammation is high (35–65%) in the chronic kidney disease (CKD) and end-stage renal disease (ESRD) populations [4–7]. Inflammation, as assessed by using C-reactive protein (CRP) level, is a strong predictor of all-cause and cardiovascular mortality in ESRD patients [5–9]. Multiple clinical factors and intercurrent clinical events may contribute to inflammation [10–14]. Studies analyzing the relationship between TCs and CRP levels have reported contradictory findings. Although several small studies reported an association between TCs and elevated CRP levels and observed a substantial reduction in CRP after TC removal with change of access to an AVF, these findings were not reproduced in data collected for 1,826 prevalent HD patients enrolled in the Hemodialysis (HEMO) Study [15–20]. In fact, in the HEMO study, higher CRP levels were inversely associated with TC use. The HEMO study investigators did not find a significant change in CRP when access was changed from a TC to an AVF or vice versa [20]. There are limited data on the longitudinal serial CRP values and the relationship to the vascular access type in an incident HD population and on the contribution of noninfected arteriovenous grafts (AVG) to inflammation. Furthermore, limited data exist for other inflammatory cytokines, such as interleukin-6 (IL-6) and interferon-*γ*-induced protein (IP-10). To investigate the role of the vascular access type on serial inflammatory cytokines we studied a longitudinal cohort of incident HD patients.

## 2. Subjects and Methods

This is a prospective, observational study in a cohort of pre-HD patients. This study was approved by the Committee of Clinical Investigation at Montefiore Medical Center, (Protocol number 06-07-336). Patients with Stage-4 and -5 chronic kidney diseases (CKD) receiving their initial HD vascular access insertion were invited to participate. Patients were recruited from 2 adult outpatient renal clinics, 8 private nephrology practices, and the inpatient renal patient population at Montefiore Medical Center, Bronx, NY. Exclusion criteria included the presence of an active inflammatory state: infection, malignancy, connective tissue disease, HIV infection, organ transplant recipient, and current use of immune modulating agents. The type of vascular access to be inserted was determined by the patient's physician. Patient data that were recorded included age, race, sex, etiology of CKD, body surface area, active tobacco use, diabetes mellitus (DM), and a history of atherosclerotic diseases including cardiovascular disease, myocardial infarction (MI), congestive heart failure (CHF), peripheral vascular disease (PVD), and cerebral vascular accident (CVA). A subjective global assessment (SGA) was calculated for each patient at time of entry into the study [21]. The conventional SGA is a three-tiered, semiquantitative scoring system based on the history and physical examination and is commonly used in nephrology to quantify the degree of malnutrition. All medications at time of randomization were documented and include the use of angiotensin-converting enzyme inhibitors (ACEI), angiotensin receptor blockers (ARB), aspirin (ASA), nonsteroidal anti-inflammatory agents (NSAID), erythropoiesis-stimulating agents (ESA), vitamin D supplements, beta-blockers, insulin, and the cholesterol-lowering statins. The date of HD initiation, access thrombosis, interventional procedures, and access survival were noted. Clinical events were recorded, including infections, hospitalizations, and patient death. 

Blood samples were drawn at baseline (within 1 month prior to access surgery), then postsurgery on days 7, 30, 90, 180, 270, 365. In patients who initiated HD, samples were drawn pre-HD. Samples were immediately centrifuged (3000 rpm for 10 minutes) and stored at −70 degrees. All cytokine assays were performed at Vanderbilt University Medical Center using cytometric bead array (Becton DickinsonTM, San Diego, Calif). The detection limits were as follows: CRP, 0.124 mg/mL; IL-6, <2 pg/mL; IP-10, 2.7 pg/mL. The intra-assay coefficient of variation (CV) for CRP was 6%, IL-6 was 7.7%, and for IP-10 was 6.2%.

## 3. Statistical Analysis

Baseline laboratory data are presented as mean ± S.D, and all cytokine values are presented as the median and interquartile range (25th to 75th percentile). Univariate analyses were performed using ANOVA (with Bonferroni correction) or Chi-square analyses where appropriate. All cytokine values were analyzed using nonparametric statistical testing, namely, the Kruskal-Wallis test. Patients who receive two concomitant vascular accesses initially (TC/AVF, or TC/AVG) were included in the TC group for all tables, figures, and analyses. Significance was determined at *P* = 0.05 (2-tailed). 

Multivariate analyses for repeated measures were performed using mixed effects models, with CRP, IL-6, and IP-10 as the dependent variables, and all were log transformed. Covariates that were included in the model (selected a priori) were age, sex, race, vascular access type, HD initiation, infection, vascular access thrombosis, cardiovascular disease, DM, and time period after vascular access surgery. Patients who had a TC and a second arteriovenous access (arteriovenous fistula [AVF] or graft [AVG]) were classified as being in the TC group for the mixed effects models. A sensitivity analysis was performed to compare the effect of a TC alone versus an AVF or AVG on inflammation (CRP, IL-6, IP-10).

## 4. Results

The study period was from August 2006 until April 2008. Of the 79 patients who initially consented to participate in the study, 14 patients did not show up for access surgery, and 1 patient withdrew from the study 1 week after access surgery. The mean followup for the remaining 64 patients was 10 months (range 0.25–12 months). 

The baseline patient demographic data are provided in Table 1. The mean patient age was 61 years, and 52% were women. The racial distribution of the study population was 48% African American, 39% Hispanic, 6% Caucasian, and 6% other race. The incidence of comorbid illnesses was: DM 69%, HTN 98%, CHF 38%, MI 17%, CVA 14%, PVD 11%, hyperlipidemia 67%. There was a history of tobacco use in 28% (active use, 5%). The mean BMI was 29.2. The etiology of ESRD was DM 48%, HTN 17%, unknown 16%, and polycystic kidney disease 6%, representative of the general ESRD population in the United States. 

The number of patients in each vascular access group was as follows: AVF, *n* = 14; AVG, *n* = 10; TC, *n* = 40 (24 with a TC only, 11 with concomitant TC and AVF placement, and 5 with both TC and AVG placement). In the AVF group, there was a significantly higher representation of men, and patients were of younger age, relative to the AVG and TC groups. There were no other significant differences in baseline demographics between access groups.

Table 2 provides baseline laboratory data, and Table 3 lists the medications upon study entry. CRP, IL-6, and IP-10 levels were significantly higher at baseline in the patients with a TC or AVG compared to patients with an AVF. None of the other baseline laboratory values differed between the access groups. Patients in the AVG group had the highest use of ASA and ESAs. Seven deaths occurred during the study period. In those 7 patients the initial vascular access and cause of death were as follows: AVF group (1 cardiac), AVG (2 sepsis, 1 cardiac death, 1 pneumonia), TC (1 sepsis, 1 pneumonia). 

There were 9 patients whose initial vascular access was a TC with a developing AVF, who subsequently had the TC removed once the AVF was useable for HD. Although the median CRP values declined after TC removal, this did not achieve statistical significance (TC/AVF: CRP 8.35 mg/L ± 15.0, versus AVF alone: 3.16 mg/L ± 1.8, *P* = 0.53). CRP data were available for only 2 patients whose initial vascular access was an AVF who then required a TC (AVF: 13.5 mg/L versus TC/AVF: 7.7 mg/L). (Data were insufficient for analysis.)

## 5. Multivariate Analyses

Mixed effects models (Tables 4, 5, and 6) were performed for CRP, IL-6, and IP-10, adjusting for the following covariates: access type, coronary artery disease, sex, age, race, HD initiation, diabetes mellitus, infection, access thrombosis, and number of days after access surgery. The adjusted models take into account every cytokine measurement and the corresponding vascular access type for each available period. The presence of a TC was a significant predictor of an elevated CRP (*P* = 0.03) and IP-10 (0.03). IL-6 levels also positively correlated with a TC, although this did not reach statistical significance, (*P* = 0.07). The presence of an AVG also significantly correlated with an elevated CRP (*P* = 0.01) and with IP-10 (*P* = 0.07), although the latter did not reach statistical significance.

Additional predictors of an elevated CRP were a history of CAD (*P* < 0.001), female sex (*P* = 0.04), and the presence of infection (*P* = 0.02). Levels of CRP decrease over time, with the highest value at 7 days after insertion compared to the CRP values at 365 days (*P* = 0.02). IL-6 levels significantly correlated with infection (*P* = 0.02), and IP-10 levels were directly associated with diabetes mellitus (0.02), male sex (0.01), and Hispanic ethnicity (0.04).

To compare the impact of TCs with other types of vascular access on inflammation, we performed a sensitivity analysis in patients with a TC exclusively (*n* = 24) and compared them to the combined group of AVF/AVG (*n* = 24). TCs remained a predictor of a higher CRP level, (estimate = 0.29, *P* = 0.055). There was no significant association with IL-6 and IP 10 levels in the sensitivity analysis.

## 6. Discussion

This study is unique in that baseline cytokine values were obtained before first access placement and prior to HD initiation, which permitted assessing the degree of preexisting inflammation for patients in each vascular access group and comparing baseline cytokine values to those obtained at multiple time periods after access insertion. The prospective study design allowed for recording all intercurrent events, which were included in the adjusted analysis. Because patients were enrolled upon initial access placement, we were able to exclude the problem of preexisting inflammation related to abandoned accesses. Finally, the association of elevated CRP levels after initial AVG insertion adds to the relative paucity of data on AVGs and inflammation in the previously existing literature.

The findings of our study may also shade light on discrepancies between the HEMO and other studies. First, the Hemodialysis study (HEMO) included only patients who were on HD therapy longer than 3 months, whereas our data and others included incident HD patients. [18, 20] We observed, in an adjusted analysis, that CRP levels are significantly elevated only in the first week following access surgery, but, similar to the HEMO study, CRP levels were not elevated at 1 month or other points throughout the following year. Secondly, in the HEMO study, AVFs and AVGs were combined into one “AV access” group for the analysis, which may have obscured differences between the AV access and TC group and differences within the AV access group (AVFs versus AVGs). We observed significantly higher CRP values associated with AVGs, not present with AVFs. 

Our data are consistent with those of Goldstein et al. who measured CRP in 73 incident HD patients (50 noninfected TCs and 23 AVFs). The median CRPs at initiation of HD were significantly higher in the TC group (44 mg/L) versus the AVF group (5 mg/L) (*P* < 0.001) [18]. Unfortunately, patients with AVGs were not included in this study. Other criticisms of this study were that only 2 CRP measurements were taken over a 6-month period, other inflammatory markers were not measured, and information about comorbid illnesses was lacking [22]. Snaedal et al. recently reported a strong association of comorbidity and clinical events with inflammation in HD patients [23]. This was a 3-month observational cohort study in prevalent HD patients. Similar to the findings of the current study, these investigators reported that a comorbidity score (including ischemic heart disease) was among those factors that were significantly associated with variations in the CRP. They did not find a significant association between vascular access type and CRP in this prevalent HD population; however as was the case in the HEMO study, AVF and AVGs were combined into the same access group for the analysis. 

There was a significant direct association between female sex and elevated CRP values in the present study, which was observed in a multivariate model adjusting for differences in vascular access type. This is in contradistinction to the findings reported by Snaedal et al. in which female sex was associated with lower CRP values, also a multivariate model [23]. A third study by Goldstein et al. reported that CRP values did not differ between men and women [18]. The relationship between gender and CRP levels is unclear and requires further investigation. 

Snaedel et al. reported widely fluctuating CRP values in 68% of patients, indicating that frequent serial CRP measurements are superior to a single measurement [23]. These authors also studied other inflammatory cytokines, including plasma tumor necrosis factor-*α* (TNF-*α*) and interleukin-10 (IL-10), which were not found to be significant predictors of outcome. 

A significant association exists between elevated proinflammatory cytokines and the presence of an AVG. We found significantly higher CRP levels associated with the presence of AVGs (estimate = 0.47, *P* < 0.001) and with TCs (estimate = 0.26, *P* = 0.03) relative to AVFs, in an adjusted analysis. These results differ somewhat with those previously reported by Movilli et al. [16] in that in our cohort of incident HD patients CRP was highest in AVG patients. In Movilli's study, which included a cohort of infection-free prevalent HD patients, TCs were associated with the highest CRP values, although both TCs (estimate = 0.88, *P* = 0.0001) and AVGs (estimate = 0.26, *P* = 0.043) were associated with significantly higher CRP levels than AVFs (adjusted analysis) [16]. It is possible that “new” AVGs placed in incident HD patients are associated with a period of peak inflammation which dampens over time as the AVG endothelializes, as was observed in the prevalent HD cohort. 

The three-step hierarchy of access-associated inflammation (TC > AVG > AVF) reported in the Movilli study closely mimics the relationship of mortality risk and vascular access type in the CHOICE study [2, 15]. The choices for healthy outcomes in caring for ESRD (CHOICE) study group reported an association of mortality and vascular access type in a cohort of incident HD patients. In this study, the adjusted relative hazard of death was 1.5 (95% CI, 1.0 to 2.2) in patients using a TC and 1.2 (95% CI, 0.8 to 1.8) in those with an AVG, relative to patients with an AVF [2]. A recent study by Perl et al. also reported an adjusted, time-dependent, relative hazard of death of 1.8 (95% CI, 1.6–1.9) in incident HD patients with a TC compared to PD patients or HD patients with an AVF/AVG [3]. As with the HEMO study, because AVG and AVF are combined, the exclusive effect of AVGs with 1-year mortality is obscured. Short of a randomized controlled trial, one may conclude that it is possible that vascular access-induced inflammation, independent of comorbidities and intercurrent illnesses, may be contributing to the excess mortality reported in TC and AVG patients in the first months of HD. 

We also report that CKD patients whose future initial vascular access is a TC or AVG have preexisting heightened inflammatory state characterized by higher baseline CRP levels, which is present prior to access surgery and HD initiation, relative to those receiving AVFs. This was found despite efforts to exclude patients from the study with preexisting inflammatory states. Patients in the TC and AVG groups were older and consisted of more female and diabetic patients. Furthermore, the subjective global assessment (SGA) scores were higher at baseline in the TC and AVG groups. Patients in the AVG group were also more anemic and had more ESA use before access, possible reflecting a chronic inflammatory state. Elevated baseline CRP values that are present in AVF and TC patients may have influenced the CRP levels observed in these groups during the follow-up period. Ortega et al. studied CRP values in 66 pre-HD patients who were followed up for 1 year [4]. The baseline CRP was 8.3 mg/L, and 35% of patients had an elevated CRP level (>6 mg/L). High CRP levels at baseline were predictive of a constant inflammatory state at followup. 

Although an association of TC use and higher ESA requirements was reported by Goldstein et al. in a small cohort (*n* = 50), more recent data reported by the dialysis outcomes and practice patterns study (DOPPS) in November 2010 did not find a significant association between catheter use and ESA dosing in over 2,500 patients, although there was a significant association between higher CRP levels and greater ESA dosing (presented at the American Society of Nephrology Renal Week 2010). Data exploring the association between ESA and TC use is not available in the present study. 

Lastly, dialysis vintage has not been shown to correlate with inflammation in two previous studies or in the present study [18, 23]. 

The major limitations of this study are the small size of the trial and the limited follow-up period of 1 year. The study was not powered using mortality as an outcome. Due to the relative low mortality rate in our cohort, associations between baseline cytokine values and mortality were not observed. We attribute the low mortality due to the predetermined inclusion criteria for this study. In an attempt to isolate the HD access from other potential mediators of inflammation we chose a select patient population, excluding unstable patients and those with active infection, autoimmune diseases, use of immune modulating medications, and malignancy. A large study in an incident pre-HD population, with a longer follow-up period, is needed to better elucidate relationships between the contribution of individual HD vascular access types to noninfectious inflammation and possible excess mortality.

## Figures and Tables

**Table 1:** Patient demographic data. All cytokine values (CRP, IL-6 and IP-10) are reported as the median, and all other lab data are presented as the means ± S.D. Patients who received both a TC and an AVF or AVG concomitantly were included in the TC group. SGA: subjective global assessment, BMI: body mass index; DM: diabetes mellitus; HTN: hypertension; CHF: congestive heart failure; MI: myocardial infarction; CVA: cerebral vascular accident; PVD: peripheral vascular disease; ESRD: end stage renal disease; GN: glomerulonephritis; PCKD: polycystic kidney disease.

	TC	AVF	AVG	*P* value
*N*	40	14	10	
Age in years	60	55	71	0.01
	(±12)	(±11)	(±12)	
Female sex	68%	14%	80%	0.005
Race/ethnicity				
Black	46%	36%	70%	ns
Hispanic	37%	57%	20%	
Caucasian	12%	0	0	
Other	5%	7%	10%	
Co-morbid Diseases				
HTN	96%	100%	100%	ns
DM	64%	50%	100%	ns
CHF	40%	29%	40%	ns
MI	12%	29%	20%	ns
CVA	20%	7%	30%	ns
PVD	16%	0	10%	ns
Hyperlipidemia	56%	64%	90%	ns
BMI	28.0	28.6	31.0	ns
	(±8.8)	(±12.1)	(±6.5)	
SGA score				ns
Low	28%	29%	0	
Medium	36%	57%	60%	
High	36%	14%	40%	
Etiology of ESRD				ns
DM	44%	36%	70%	
HTN	12%	29%	30%	
GN	0%	14%	0	
PCKD	8%	7%	0	
Other	29%	14%	0	
Past malignancy	4%	7%	20%	ns
Tobacco use				ns
Current	4%	14%	0	
Former	32%	21%	10%	

**Table 2:** Baseline laboratory data. All values are presented as the means ± S.D. eGFR: estimated glomerular filtration rate; PTH: parathyroid hormone; Hgb: hemoglobin, LDL: low-density lipoprotein.

	TC	AVF	AVG	*P* value
CRP (median, mg/L)	7.2	1.3	13.9	0.035
CRP >5 mg/L (%)	54%	23%	75%	0.04
IL-6 (median, pg/mL)	16.5	11.4	12.2	0.06
IP-10 (median, pg/mL)	222	148	289	ns
GFR (mL/min)	10	14	11	ns
	(±7)	(±6)	(±4)	
Albumin (g/dL)	3.5	3.7	3.3	ns
	(±0.5)	(±0.5)	(±0.6)	
Phosphorous (mg/dL)	6.1	5.1	5.6	ns
	(±1.9)	(±1.2)	(±1.4)	
Calcium (mg/dL)	8.0	8.5	8.7	ns
	(±1.3)	(−0.7)	(±0.7)	
PTH (pg/mL)	710	358	256	ns
	(±990)	(±283)	(±134)	
Hgb (g/dL)	9.1	10.4	10.1	0.009
	(±1.5)	(±1.0)	(±2.0)	
Ferritin (ng/mL)	328	250	255	ns
	(±325)	(±250)	(±225)	
Total cholesterol (mg/dL)	180	161	184	ns
	(±56)	(±67)	(±39)	
LDL cholesterol (mg/dL)	104	85	94	ns
	(±47)	(±51)	(±26)	
Hepatitis C Ab	13%	14%	10%	ns

**Table 3:** Medications upon study entry. ASA: aspirin; ACEI/ARB: angiotensin converting enzyme inhibitor or angiotensin receptor blocker; ESA: erythropoietin stimulating agent.

	TC	AVF	AVG	*P* value
Statin	55%	50%	70%	ns
ASA	28%	64%	70%	0.009
Beta-blocker	70%	79%	80%	ns
Insulin	48%	43%	80%	ns
Oral hypoglycemic	35%	0	20%	0.03
Phosphate binder	33%	21%	30%	ns
Vitamin D	43%	43%	50%	ns
ACEI/ARB	28%	50%	60%	ns
ESA	38%	29%	90%	0.004

**Table 4:** Mixed-effects model for CRP (log transformation) including all time points throughout the study, (*n* = 662). In an adjusted analysis, CRP levels positively correlated with the presence of a TC or AVG, history of coronary artery disease (CAD), and the time period 7 days after hemodialysis access insertion. There was an inverse correlation of CRP with male sex, and absence of infection. CRP: C-reactive protein, TC: tunneled catheter, AVG: arteriovenous graft, AVF: arteriovenous fistula, DM: diabetes mellitus, CAD: coronary artery disease, HD: hemodialysis. Definitions: CRP: C-reactive protein, TC: tunneled catheter, AVG: arteriovenous graft, AVF: arteriovenous fistula, DM: diabetes mellitus, CAD: coronary artery disease, HD: hemodialysis.

Parameter	Estimate	95% confidence interval		*P*
		Lower	Upper	
Access type				
AVF	Ref.			
TC	0.26	0.03	0.49	0.03
AVG	0.47	0.14	0.81	0.01
CAD (no)	−0.54	−0.89	−0.19	<0.001
Sex (male)	−0.28	−0.56	−0.01	0.04
Infection (no)	−0.30	−0.55	−0.06	0.02
Age	0.00	−0.01	0.01	0.91
Race				
Hispanic	Ref.			
African American	0.16	−0.11	0.43	0.23
Caucasian	−0.11	−0.65	0.43	0.69
DM (no)	−0.08	−0.36	0.21	0.60
HD (no)	0.01	−0.13	0.15	0.89
Access thrombosis (no)	−0.08	−0.35	0.19	0.57
Number of days after access insertion				
7 days	0.32	0.05	0.60	0.02
30 days	−0.14	−0.40	0.11	0.26
90 days	−0.09	−0.35	0.17	0.50
180 days	−0.19	−0.43	0.06	0.13
270 days	−0.06	−0.27	0.15	0.58
365 days	Ref.			

**Table 5:** Mixed-effects model for IL-6 (log transformation) including all time points throughout the study (*n* = 662). In an adjusted analysis, IL-6 levels positively correlated with the presence of a TC, although this did not reach statistical significance (*P* = 0.07). There was an inverse correlation of IL-6 with absence of infection. IL-6: interleukin 6, TC: tunneled catheter, AVG: arteriovenous graft, AVF: arteriovenous fistula, DM: diabetes mellitus, CAD: coronary artery disease, HD: hemodialysis

Parameter	Estimate	95% confidence interval		*P*
		Lower	Upper	
Access type				
AVF	Ref.			
TC	0.15	−0.01	0.32	0.07
AVG	0.12	−0.10	0.35	0.28
CAD (no)	−0.17	−0.38	0.05	0.14
Sex (male)	0.08	−0.08	0.25	0.33
Infection (no)	−0.24	−0.44	−0.04	0.02
Age	0.00	0.00	0.01	0.67
Race				
Hispanic	Ref.			
African American	0.10	−0.06	0.27	0.21
Caucasian	0.02	−0.30	0.34	0.89
DM (no)	−0.08	−0.25	0.08	0.32
HD (no)	−0.01	−0.13	0.10	0.85
Access thrombosis (no)	0.09	−0.09	0.26	0.35
Number of days after access insertion				
7 days	0.05	−0.16	0.27	0.61
30 days	−0.12	−0.33	0.09	0.25
90 days	−0.16	−0.37	0.04	0.12
180 days	−0.24	−0.43	−0.05	0.01
270 days	−0.14	−0.31	0.02	0.09
365 days	Ref.			

**Table 6:** Mixed-effects model for IP-10 (log transformation) including all time points throughout the study (*n* = 662). In an adjusted analysis, IP-10 levels positively correlated with the presence of a TC and AVG, although the latter did reach statistical significance, (*P* = 0.07). IP-10 levels were also significantly associated with male sex. There was an inverse correlation of IP-10 with DM and African American or Caucasian race. IP-10: interferon-*γ*-induced protein, TC: tunneled catheter, AVG: arteriovenous graft, AVF: arteriovenous fistula, DM: diabetes mellitus, CAD: coronary artery disease, HD: hemodialysis

Parameter	Estimate	95% Confidence Interval		*P*
		Lower	Upper	
Access type				
AVF	Ref.			
TC	0.26	0.03	0.49	0.03
AVG	0.30	−0.03	0.63	0.07
CAD (no)	0.17	−0.24	0.58	0.41
Sex (male)	0.43	0.12	0.74	0.01
Age	0.000	−0.01	0.01	0.65
Race				
Hispanic	Ref.			
African American	−0.30	−0.60	0.01	0.01
Caucasian	−0.64	−1.25	−0.04	0.04
DM (no)	−0.38	−0.70	−0.05	0.02
Infection (no)	−0.14	−0.38	0.10	0.27
Access thrombosis (no)	0.10	−0.09	0.29	0.31
Number of days after access insertion				
7 days	0.08	−0.17	0.34	0.52
30 days	0.06	−0.19	0.32	0.64
90 days	0.02	−0.22	0.26	0.85
180 days	−0.10	−0.29	0.09	0.31
270 days	−0.05	−0.21	0.11	0.51
365 days	Ref.			
